# The Field and Limitation of the Operative Surgery of the Human Brain

**Published:** 1886-04

**Authors:** 


**The Field and Limitation of the Operative Surgery of the Human Brain.** By John B. Roberts, A. M., M. D., Professor of Anatomy and Surgery in the Philadelphia Polyclinic; Surgeon to St. Mary's Hospital. Philadelphia: P. Blakiston, Son & Co., 1012 Walnut street. 1885.

This excellent work, which has been out of press some time, has attracted considerable attention and much merited praise. It seems probable that in the near future operative interference will be often resorted to in tumors and other affections of the brain. To study the appropriate methods of procedure is very necessary in view of this fact. The creed which the Doctor follows is given:

I. The complexus of symptoms, called "compression of the brain," is due, not so much to displacing pressure exerted on the brain substance, as it is to some form or degree of inter-cranial inflammation.

II. The conversion of a closed (simple) into an open (compound) fracture by incision of the scalp is, with the improved methods of treating wounds, attended with very little increased risk to life.

III. The removal of portions of the cranium by the trephine or other cutting instruments is, if properly done, attended with but little more risk to life than amputation of a finger through the metacarpal bone.

IV. In the majority of cranial fractures, the inner table is more extensively shattered and splintered than the outer table.

V. Perforation of the cranium is to be adopted as an exploratory measure, almost as often as it is demanded for therapeutic reasons.

VI. Drainage is more essential in wounds of the brain than in wounds of other structures.

VII. Many regions of the cerebral hemispheres of man may be incised and excised with comparative impunity.

VIII. Accidental or operative injuries to the cerebral membranes, meningeal arteries, or venous sinuses, should be treated as are similar lesions of similar structures in other localities.

IX. The results of the study of cerebral localization are more necessary to the conscientious surgeon than to the neurologist. Some of these propositions are indeed radical, but we cannot deny the fact that the Doctor maintains his points in his discussions on these subject.

In 1880, June number of this Journal, Dr. S. G. Dorr, of this city, gave a report of cases of trephining, and made mention of the surgical engine, drills and burrs for removing the tables of the skull. Of this, Dr. Roberts says: "Much stress has been laid by some writers on the danger of wounding the membranes and brain with the trephine. With a conical trephine or burr of the surgical engine, as recommended by Dorr and myself, there is no danger of this." The book is well printed and published, reflecting credit on publishers as well as author.

